# DISCRIMINATION AND PSYCHOLOGICAL WELL-BEING IN LATER LIFE: LONGITUDINAL EVIDENCE FROM CHINA

**DOI:** 10.1093/geroni/igad104.2361

**Published:** 2023-12-21

**Authors:** Shuai Zhou, Xue Bai

**Affiliations:** The Hong Kong Polytechnic University, Kowloon, Hong Kong; The Hong Kong Polytechnic University, Kowloon, Hong Kong

## Abstract

Although ample evidence shows a negative association between discrimination and well-being outcomes, yet few longitudinal studies have explored the prevalence and consequences of multiple forms of discrimination among older adults. This study investigated the effects of interpersonal and institutional discrimination on psychological well-being among Chinese older adults and whether such effects were conditioned on subjective socioeconomic status. Data were retrieved from the China Family Panel Studies, consisting of 9,356 older respondents aged 45 and older in 2010 with their follow-up observations during 2012–2016. Random-effects panel regression analyses were performed to examine the relationships between multiple forms of discrimination on psychological distress and life satisfaction, separately, as well as the moderating effects of subjective socioeconomic status. Results revealed that one in four older adults experienced at least one form of discrimination during the past 12 months. Most prevalent discriminatory experiences encompassed wealth-based discrimination, delay and stalling at government agencies, and unfair treatment by government officials. Experiences of discrimination were negatively associated with life satisfaction and positively associated with psychological distress. Subjective socioeconomic status ameliorated the negative effect of interpersonal discrimination on life satisfaction. Findings reveal the detrimental effects of discrimination on psychological well-being that both interpersonal and institutional discrimination could reduce satisfaction with life and exaggerate psychological distress. Findings imply that subjective socioeconomic status could protect older adults from harmful effects of interpersonal discrimination, but not institutional discrimination.

